# Ubiquitin-like modifier 1 ligating enzyme 1 relieves cisplatin-induced premature ovarian failure by reducing endoplasmic reticulum stress in granulosa cells

**DOI:** 10.1186/s12958-022-00956-9

**Published:** 2022-05-24

**Authors:** Xiangting Tang, Hao Dong, Zhi Fang, Jingyi Li, Qi Yang, Ting Yao, Zezheng Pan

**Affiliations:** 1 Basic Medical College, Nanchang University, Nanchang, 330006 China; 2 The First Affiliated Hospital of Nanchang University, Nanchang, 330006 China

**Keywords:** UFL1, POF, ER stress, Cisplatin, Apoptosis, GCs

## Abstract

**Background:**

Ubiquitin-like modifier 1 ligating enzyme 1 (UFL1), the ligase of the UFMylation system, has recently been reported to be involved in apoptosis and endoplasmic reticulum stress (ER stress) in a variety of diseases. Premature ovarian failure (POF) is a gynecological disease that severely reduces the fertility of women, especially in female cancer patients receiving chemotherapy drugs. Whether UFL1 is involved in protection against chemotherapy-induced POF and its mechanism remain unclear.

**Methods:**

In this study, we examined the function of UFL1 in ovarian dysfunction and granulosa cell (GC) apoptosis induced by cisplatin through histological examination and cell viability analysis. We used western blotting, quantitative real-time PCR (qPCR) and immunofluorescence (IF) to detect the expression of UFL1 and the levels of ER stress specific markers. Enzyme linked immunosorbent assays were used to detect the levels of follicle-stimulating hormone (FSH) and estrogen (E_2_) in ovaries and GCs. In addition, we used infection with lentiviral particle suspensions to knock down and overexpress UFL1 in ovaries and GCs, respectively.

**Results:**

Our data showed that the expression of UFL1 was reduced in POF model ovaries, accompanied by ER stress. In vitro, cisplatin induced a stress-related increase in UFL1 expression in GCs and enhanced ER stress, which was aggravated by UFL1 knockdown and alleviated by UFL1 overexpression. Furthermore, UFL1 knockdown resulted in a decrease in ovarian follicle number, an increase in atretic follicles, and decreased expression of AMH and FSHR. Conversely, the overexpression of UFL1 reduced cisplatin-induced damage to the ovary in vitro.

**Conclusions:**

Our research indicated that UFL1 regulates cisplatin-induced ER stress and apoptosis in GCs, and participates in protection against cisplatin-induced POF, providing a potential therapeutic target for the clinical prevention of chemotherapeutic drug-induced POF.

**Graphical Abstract:**

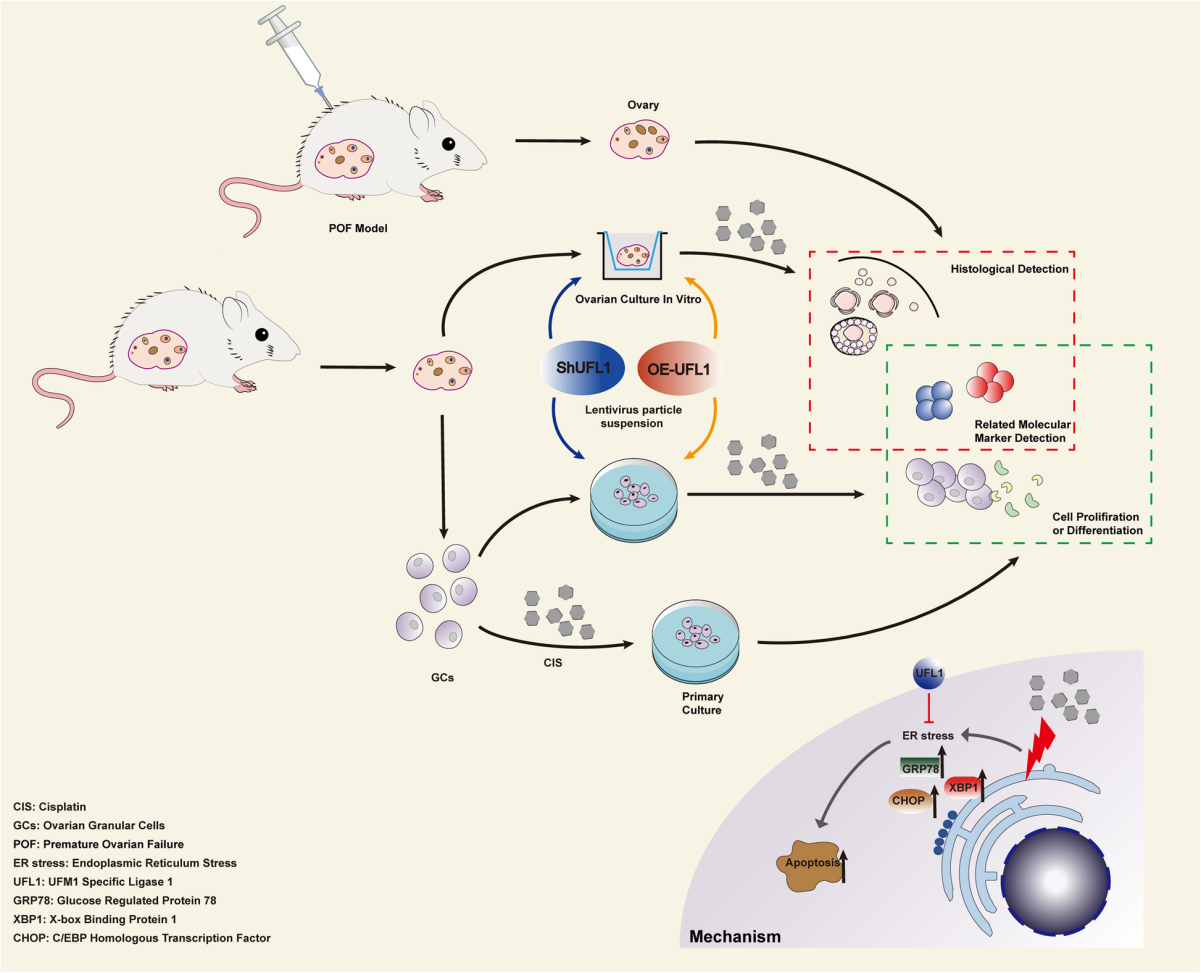

**Supplementary Information:**

The online version contains supplementary material available at 10.1186/s12958-022-00956-9.

## Introduction

Ubiquitin-like modifier 1 ligating enzyme 1 (UFL1), also known as KIAA0776, RCAD, NLBP and Maxer, has a molecular weight of approximately 90 kDa and is composed of 794 amino acids, and it is the only identified E3 ligase in the UFMylation modification system [1–3]. In addition to playing a crucial role in this ubiquitin-like system, UFL1 is also involved in various processes such as endoplasmic reticulum stress (ER stress), apoptosis, autophagy, inflammation, and oxidative stress in tissues such as the hematopoietic system, heart, breast, and small intestine [4]. Li et al. found that the loss of UFL1 weakened the protein kinase-like endoplasmic reticulum kinase (PERK) signal in the unfolded protein response (UPR) and aggravated ER stress, while the upregulation of UFL1 in cardiomyocytes could maintain ER homeostasis and prevent cardiac stress [5]. Zhang et al. discovered that UFL1 exhaustion leads to abnormal activation of transformation related protein 53 (P53) and autophagic degradation which increases cell death, finally leading to embryo damage and hematopoietic defects [6]. Cai et al. revealed that the deletion of UFL1 contributed to a large loss of intestinal Paneth cells and goblet cells, which changed the intestinal tract bacteria and caused susceptibility to enteritis [7]. Therefore, previous researches proved that UFL1 can maintain the ER homeostasis of cells and play an important role in embryonic development and certain types of disease progression. Interestingly, granulosa cell (GC) proliferation and differentiation are closely related to ER stress [8], however, whether UFL1 can influence follicular development and protect ovarian function by relieving ER stress is still unclear.

The ER is the main organelle responsible for the biosynthesis of lipids and sterols, maintaining calcium homeostasis, and managing protein synthesis, folding and secretion into other organelles [9]. However, various pathological conditions, such as hypoxia, starvation, and calcium depletion, hinder the proper folding and modification of proteins and finally trigger ER stress [9–11]. The specific markers of ER stress are Glucose Regulated Protein 78 (GRP78), the spliceosome of X-box Binding Protein 1 (XBP1s), C/EBP Homologous Transcription Factor (CHOP), the upregulation of which indicates the aggravation of ER stress [12]. The proliferation and differentiation of GCs in the follicle regulates the maturation of oocytes and impacts female reproductive function so that the occurrence of severe ER stress in GCs may lead to ovarian dysfunction and infertility [11, 12].

Premature ovarian failure (POF) is defined as an ovarian functional defect occurring before the age of 40 years that is characterized by amenorrhea, hypogonadism and estrogen deficiency [13–15]. There are several factors that cause POF. Chemotherapeutics are one of the important causes, but the molecular mechanism is unknown [16, 17]. The maturation of oocytes requires GCs to provide nutrients and growth factors [18], so GC apoptosis or damage may be the main reason for follicular atresia and POF. Studies have verified that excessive ER stress can initiate apoptotic cell death via the upregulation of the UPR transcription factor CHOP [19]. Here, we hypothesize that chemotherapeutic drugs causing GC apoptosis may activate the severe ER stress pathway. Previous studies have shown that UFL1 can regulate ER stress in cardiomyocytes and bone marrow cells [5, 6]; however, whether UFL1 can alleviate ER stress in GCs to rescue POF remains to be explored.

In this study, we explored the function of UFL1 in protecting follicles and GCs by constructing a POF model and treating GCs in primary culture with cisplatin. Our research demonstrated that UFL1 expression obviously increased all types of follicles and rescued ovarian function. The results showed that UFL1 survives cisplatin-induced ovarian GCs apoptosis by relieving ER stress and alleviates POF to some extent. Our study suggests that UFL1 may be a molecular target to relieve ovarian injury induced by chemotherapy drugs and provides a new clinical treatment strategy in the future.

## Materials and methods

### Animals and treatment

The 6 ~ 8-week-old Kunming mice used in the experiments were purchased from the Department of Animal Science of Nanchang University Jiangxi Medical College. All mice were provided abundant food and tap water and were intraperitoneally injected with cisplatin (2.5 mg/kg and 5.0 mg/kg, Sigma, USA) for 10 days to construct the POF model [20, 21]. The study was approved by the Animal Care Committee of Nanchang University Jiangxi Medical College (Animal protocol: NCDXSYDWFL-2015097).

### Primary ovarian granulosa cell isolation and culture

Primary GC was isolated from ovarian follicles and cultured as described previously [22, 23]. The ovaries were collected and GCs were isolated mechanically under aseptic conditions at 48 h after injection of 20 U pregnant mare serum gonadotropin (PMSG). The GCs were placed in a culture plate containing 10% (v/v) FBS (Gibco, Staley Rd, Grand Island, NY, USA), 100 U FSH and 1% antibiotics in DMEM/F12 1X (1:1) (Gibco, USA) and were incubated at 37 °C with 5% CO_2_ for 48 h [24]. After incubation, the appropriate number of GCs were plated on culture plates after counting using a hemocytometer according to each experimental requirement.

### Cell treatment

GCs were treated with different concentrations of cisplatin (0, 5, 10, 15 and 20 μM) for 24 h to determine the appropriate dosing concentrations. Then, GCs were treated with 20 μM cisplatin for different durations (0 h, 3 h, 6 h, 12 h, to 24 h) to determine the appropriate duration of drug treatment. To detect changes in the effect of cisplatin on ovaries or GCs caused by UFL1 overexpression or knockout, we first infected ovaries or GCs with lentivirus particles to change the expression level of UFL1, and then treated the ovaries or GCs with cisplatin. Changes after UFL1 knockdown were detected after cisplatin treatment with 20 μM cisplatin for 12 h, and changes after UFL1 overexpressing were detected after cisplatin treatment with 20 μM cisplatin for 24 h.

### Cell transfection

We collected lentivirus particle suspensions by cotransfecting pSPAX2, pVSVG and the target plasmid into 293 T cells. Lipofectamine 2000 (Invitrogen, Carlsbad, CA, USA) was used to transfect cells or ovaries according to the manufacturer’s instructions. The plasmid was extracted with glycerol broth (GenePharma, Shanghai, China) according to the manufacturer’s instructions. The amplified product was purified, the UFL1 gene was cloned into the pEX-3 vector, and the resulting vector was then transferred into competent cells. GCs were seeded into six-well plates and infected with lentivirus at a density of 60–70% for 48 h. We used two pairs of UFL1 shRNAs. One of the UFL1 shRNA sequences was 5′-GAAACACTTCTGTGTCAGAAA-3′, and the antisense sequence was 3′-GCTCTGGAACATGGGTTGATA-5′. The other sequence was 5′-GCAGCAGAAGCTTGTGATATT-3′, and the antisense sequence was 5′-TATCACAAGCTTCTGCTGCTT-3′.

### Ovary extraction and culture

The ovaries were dissociated and placed in precooled PBS solution and as much surrounding tissue was removed as possible. Ovaries were placed in a Transwell chamber (Millicell, Darmstadt, Germany) in Waymouth medium (Sigma, USA) containing 10% (v/v) FBS (Gibco, Staley Rd, Grand Island, NY, USA), 0.23 mM sodium pyruvate, and penicillin and streptomycin (P/S) (Solarbio, Beijing, China) [25]. For in vitro experiments, ovaries were cultured for 7 days with shRNA or OE-UFL1 lentivirus particle suspension, which was replaced every 24 h.

### HE staining and follicle counting

Ovaries were immediately fixed overnight with 4% paraformaldehyde at room temperature and then embedded in paraffin. Paraffin sections of the ovarian tissue were stained with hematoxylin and eosin solution (G1001 and G1004, Servicebio, Wuhan, China) according to the manufacturer's instructions to observe the pathological structure of the ovary and to calculate the number of follicles at all levels. Follicular counts were performed according to the method proposed in previous studies [24, 26]. In each group, 5 slides were randomly selected from the largest cross continuous sections of the ovarian center, 5 nonrepeating views were selected from each slide for statistical analysis, and the average value of the 5 slides was taken. Primordial follicles are nongrowing follicles that consist of an oocyte that is partially or completely encapsulated by flattened squamous pre-GCs. The primary follicle contains an oocyte surrounded by a layer of cuboid GCs. Secondary follicles contain oocytes surrounded by multiple layers of GCs. The chromosomes of oocytes contained in atretic follicles are dense and dissolved, the nuclei are shrunken, and the GCs on the follicle surface exhibit nuclear pyknosis. Granulosa cells from atretic follicules detach from the membrane layer and float in the follicle fluid or may even be fragmented [27, 28].

### Cell proliferation assay

GCs were spread on a 96-well plate at 2000 cells per well and treated with cisplatin at different concentrations (0, 5, 10, 15 and 20 μM) for 24 h or with 20 μM cisplatin for different durations (0 h, 3 h, 6 h, 12 h, to 24 h). A Cell Counting Kit (CCK8, Transgen BioTECH, Beijing, China) was used to detect cell proliferation activity. Primary GC were isolated from ovarian follicles as described previously, and cell density reached 70%-80% after 48 h of culture. After treatment with lentiviral suspension for 48 h, cells were seeded into 6-well plates (1000 cells per well) for 5 days for clone formation experiments and into 96-well plates (4000 cells per well) for 48 h for EdU staining (KGA331, KeyGEN BioTECH, China). Crystal violet (G1062, Solarbio, China) was used to stain and count the number of colonies.

### Immunoblotting, Immunohistochemistry, and Immunofluorescence

Immunoblotting (IB), immunohistochemistry (IHC), and immunofluorescence (IF) were performed as described previously [29]. Images were acquired using a NIKON Eclipse 80i microscope. The primary antibodies used in this study included beta-tubulin (10,094–1-AP, Proteintech, Wuhan, China), UFL1 (ab226216, Abcam, Cambridge, UK), XBP1 (ab37152, Abcam, Cambridge, UK), GRP78 (66,574–1-lg, Proteintech, Wuhan, China), CHOP (15,204–1-AP, Proteintech, Wuhan, China), BCL-2 (26,593–1-AP, Proteintech, Wuhan, China), BAX (WL01637, Wanleibio, Shenyang, China), Caspase3/cleaved-Caspase3 (WL02117, Wanleibio, Shenyang, China), Caspase-3 (WL04004, Wanleibio, Shenyang, China), AMH (HA500137, HUABIO, Hangzhou, China), and FSHR (22,665–1-AP, Proteintech, Wuhan, China). All HRP- and fluorophore-conjugated secondary antibodies were obtained from Elabscience. The EdU kit was purchased from Keygen BioTECH (KGA337-1000).

### Quantitative real-time PCR

Total mRNA was extracted from tissue or cell samples using TRIzol reagent. cDNA was obtained by reverse transcription of mRNA according to the instructions of the PrimeScript RT kit for subsequent testing. TB Green Mix was used for real-time quantitative PCR. The reference gene was β-actin. The PCR primer sequences are shown in Table 1 and were used only for PCR amplification of specific segments of the gene of interest.

**Table 1:** Sequences used for quantitative real-time PCR

Gene Name	Primer Sequence: 5’-3’	Gene ID
UFL1	Forward: TGGCTATCTAGAATTTGACGCT	NM_001355512.1
	Reverse: CATAGCACATCTTCAACTGAC	
GRP78	Forward: ATGATGAAGTTCATGTGGTGG	NM_001163434.1
	Reverse: CTGATCGTTGGCTATGATCTCC	
ATF4	Forward: AGTTTAGAGCTAGGCATGAAG	NM_001287180.1
	Reverse: CATACAGATGCCATGTCATTG	
CHOP	Forward: CTCGCTCTCCAGATTCCAGTC	NM_001290183.1
	Reverse: CTTCATGCGTTGCTTCCCA	
ACTB	Forward: CTACCTCATGAGATCCTGACC	NM_007393.5
	Reverse: CACAGCTTCTCTTTGATGTCAC	

### Mitochondrial membrane potential measurement

Tetramethylrhodamine ethyl ester (TMRE) (ThermoFisher Scientific, MA, USA) was used to detect mitochondrial membrane potential (MMP). GCs were washed three times in sterile PBS, and TMRE working liquid was added to each well and incubated for 30 min under 5% CO_2_ at 37 °C conditions. GCs were then washed with PBS and analyzed on a flow cytometer according to the manufacturer’s instructions.

### Hormone measurement with enzyme-linked immunosorbent assays

For serum sample collection, at the end of the experiment, a blood sample was collected from the eyeball vein and centrifuged at 3000 rpm for 15 min. For in vitro cultured ovarian tissue samples and granulosa cells, ovarian tissue homogenate and GC suspension homogenate were collected after the required experimental treatments. The FSH (E-EL-M0511c, Elabscience, Wuhan, China) or AMH (E-EL-M3015, Elabscience, Wuhan, China) levels in the samples were measured using ELISA kits according to the manufacturer's instructions.

### Statistical analysis

Statistical analysis was performed using GraphPad Prism 8 software, and one-way analysis of variance was used to detect significant differences between multiple sets of data. *P* values < 0.05 were considered statistically significant. All data are expressed as the mean ± standard error of at least three independent experiments.

## Results

### The expression of UFL1 decreases in POF model mouse ovaries

**Fig. 1:**
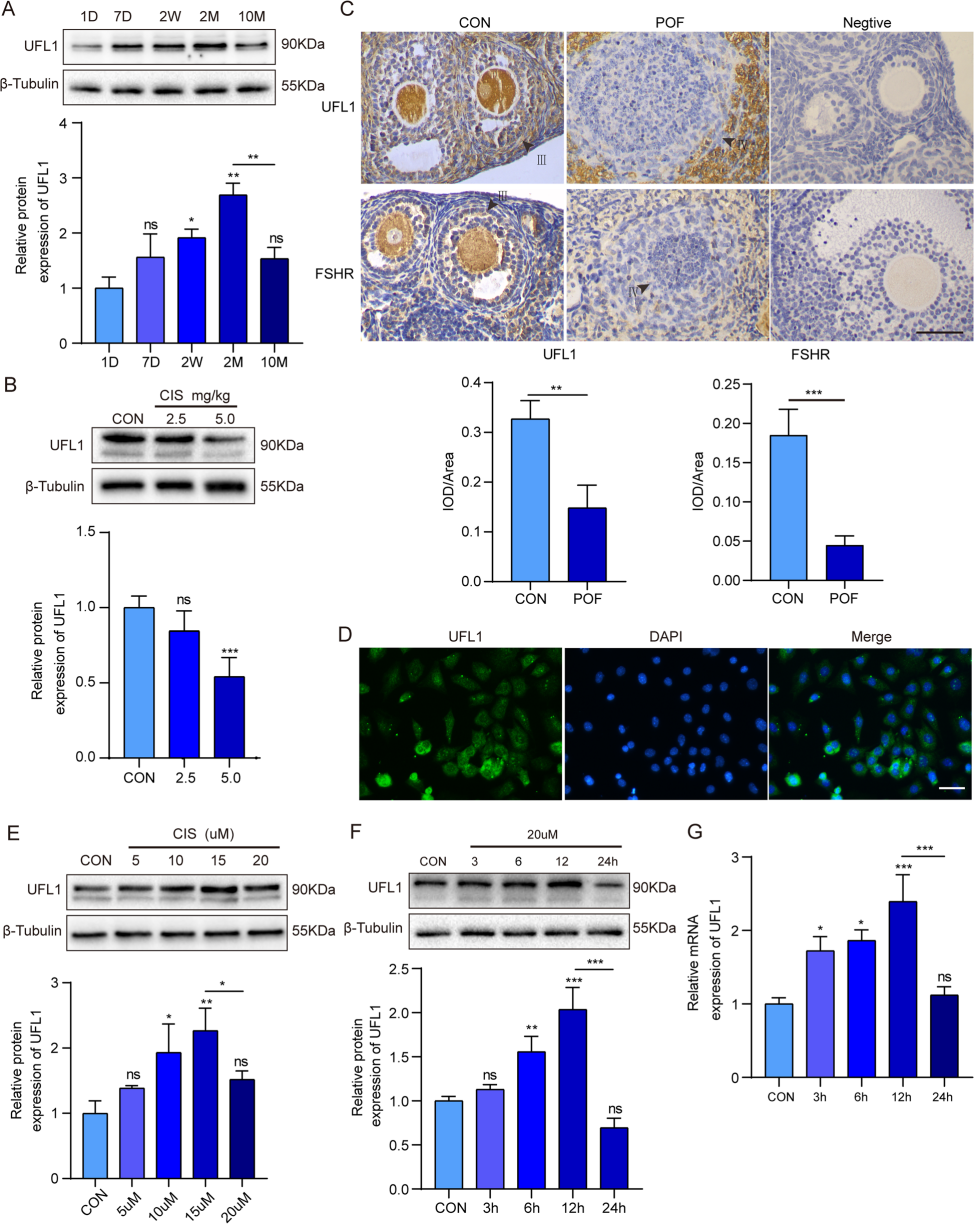
The expression of UFL1 decreased in POF model mouse ovaries and GCs. **A** The protein level of UFL1 in mouse ovaries at 1D, 7D, 2 W, 2 M, and 10 M. **B** The UFL1 and FSHR protein level in POF ovaries. **C** IHC to detect UFL1 in POF ovaries. Bar, 20 μm. Primordial follicle (I), primary follicle (II), secondary follicle (III), atretic follicle (IV). **D** IF to detect UFL1 in primary GCs. Bar, 20 μm. **E** The level of UFL1 in GCs treated with different concentrations of cisplatin. **F, G** The protein and mRNA levels of UFL1 in GCs treated with 20 μM cisplatin for different durations. *n* ≥ 3 for each group. * *p* < 0.05; ** *p* < 0.01; *** *p* < 0.001 compared with the control group

First, we identified the expression of UFL1 in ovaries at different stages of development. During mouse development from 1D to 10 M, the expression of UFL1 increased from 1D to 2 M and tended to decrease at 10 M at the protein and mRNA levels (*p* < 0.05) (Fig. 1A and Figure S1A), indicating that the abundance of UFL1 is associated with ovarian aging. We constructed mouse models of POF by intraperitoneal injection of cisplatin [20, 21] (Figure S1B-E, S1H-I). UFL1 protein expression was significantly decreased in the ovaries of POF model mice (Fig. 1B). Immunohistochemical analysis with FSHR, a specific marker of GCs, and UFL1 showed that the decreased expression of UFL1 protein occurred mainly in GCs (*p* < 0.01) (Fig. 1C). Therefore, we isolated primary ovarian GCs (Figure S1F-G) and determined the expression and localization of UFL1 by IF in GCs (Fig. 1D). Next, we treated GCs with different concentrations of cisplatin for 24 h, and the results revealed that the protein expression of UFL1 increased within a certain cisplatin concentration range (< 15 μM) but showed a decrease at 20 μM (*p* < 0.05) (Fig. 1E). Meanwhile, the UFL1 protein level was elevated at 12 h and reduced at 24 h after treatment with 20 μM cisplatin (*p* < 0.001) (Fig. 1F, G). In short, the expression of UFL1 was weakened in POF ovaries, instantaneously upregulated under stress and eventually decreased in GCs under cisplatin treatment.

### Cisplatin treatment triggers ER stress in GCs and ovaries

**Fig. 2:**
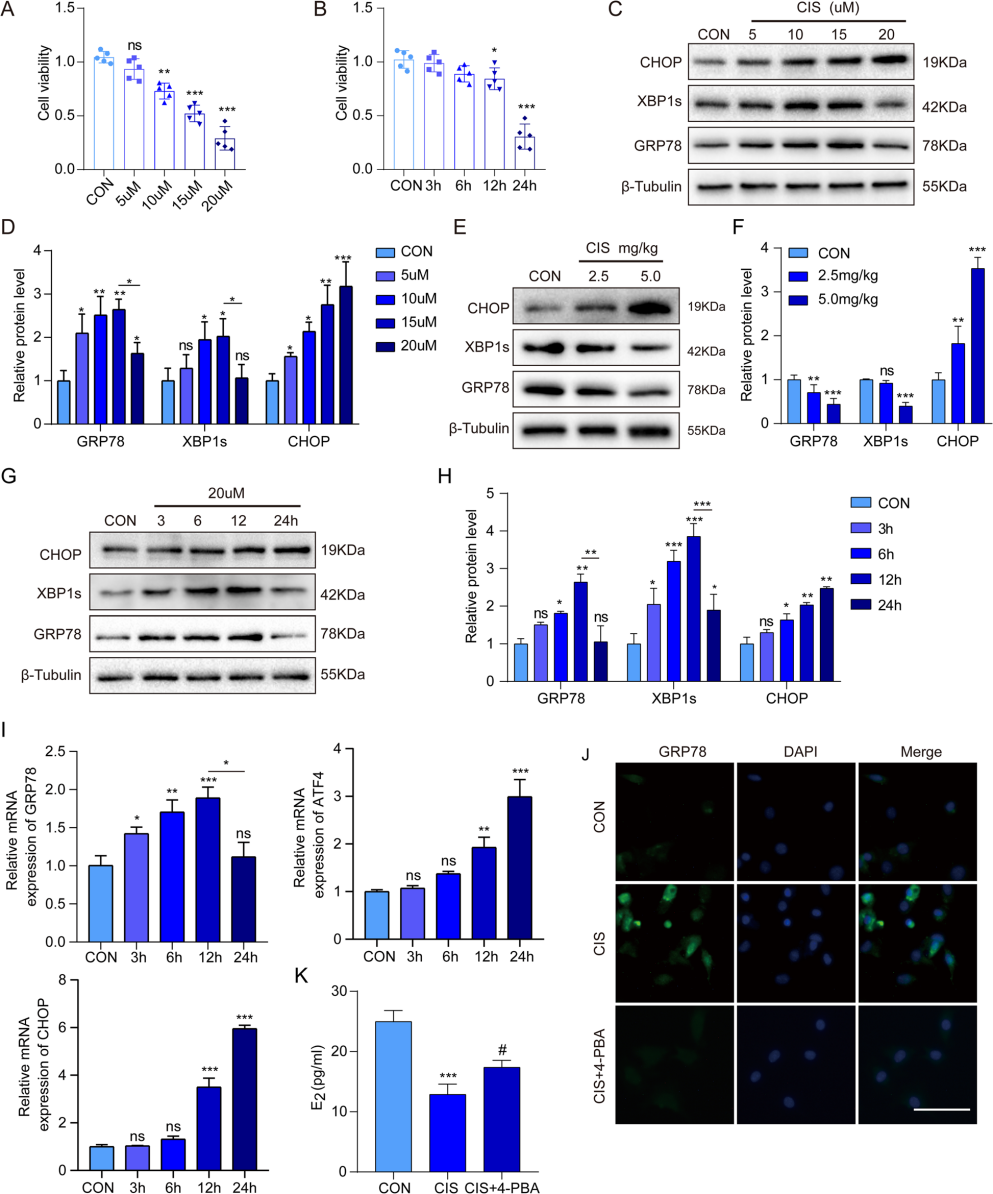
Cisplatin treatment reduced GC cell viability and induced adaptive ER stress in GCs and ovaries. **A** The viability of GCs treated with different concentrations of cisplatin. **B **CCK-8 analysis detected the viability of GCs treated with 20 μM cisplatin for different durations. **C-D** The protein levels of GRP78, XBP1s and CHOP in GCs treated with different concentrations of cisplatin. **E–F** The protein levels of GRP78, XBP1s and CHOP in POF ovaries. **G-H** The protein levels of GRP78, XBP1s and CHOP in GCs at different time points under 20 μM cisplatin treatment. **I** The mRNA levels of GRP78, ATF4 and CHOP in GCs at different time points under 20 μM cisplatin treatment. **J** IF to detect the expression of GRP78 in GCs in the control group, cisplatin treatment group, and 4-PBA cotreatment group. Bar, 50 μm. **K** ELISA to detect the E_2_ levels in GCs in the control group, cisplatin treatment group, and 4-PBA cotreatment group. *n* ≥ 3 for each group. * *p* < 0.05; ** *p* < 0.01; *** *p* < 0.001 compared with the control group. # *p* < 0.05 compared with the cisplatin treatment group

To observe the damaging effect of cisplatin on GCs, CCK-8 analysis was used to detect the viability of GCs. The results showed that cell proliferation was inhibited by cisplatin in a time- and gradient- dependent manner (Fig. 2A, B). We next determined the occurrence of ER stress in POF GCs and ovaries. When treated with different concentrations of cisplatin for 24 h, the protein expression of ER stress specific markers GRP78 and XBP1s in GCs increased with 15 μM and decreased at 20 μM cisplatin (*p* < 0.05), while CHOP was persistently increased (Fig. 2C, D). As shown in Fig. 2E and 2F, the changes in UFL1 and ER stress-specific markers in GCs treated with high concentrations of cisplatin (20 μM) for 24 h were similar to those observed in POF ovaries. Then, we chose 20 μM cisplatin to analyze the changes in ERs markers in GCs at different time points, and the data revealed that the levels of GRP78 and XBP1s increased within 12 h and was weakened after 24 h (*p* < 0.05), while CHOP increased slightly within 12 h and was upregulated significantly after 24 h (Fig. 2G-I). Furthermore, we explored whether alleviating ER stress could reduce the damage of cisplatin to GCs. IF showed that the fluorescence intensity of the GRP78 protein increased after cisplatin treatment and was inhibited by 4-phenylbutyric acid (4-PBA, an ER stress inhibitor) (Fig. 2J). Meanwhile, the level of estrogen (E_2_) secreted by GC in the 4-PBA-treated group was higher than that of the cisplatin-treated group (Fig. 2K), suggesting that inhibiting ER stress may be helpful to alleviate GC damage. In summary, cisplatin induced time- and concentration-dependent ER stress in the ovary and GCs, and the functional damage to GCs was ameliorated by inhibiting ER stress.

### UFL1 deficiency aggravates cisplatin-induced ER stress and apoptosis in GCs

**Fig. 3:**
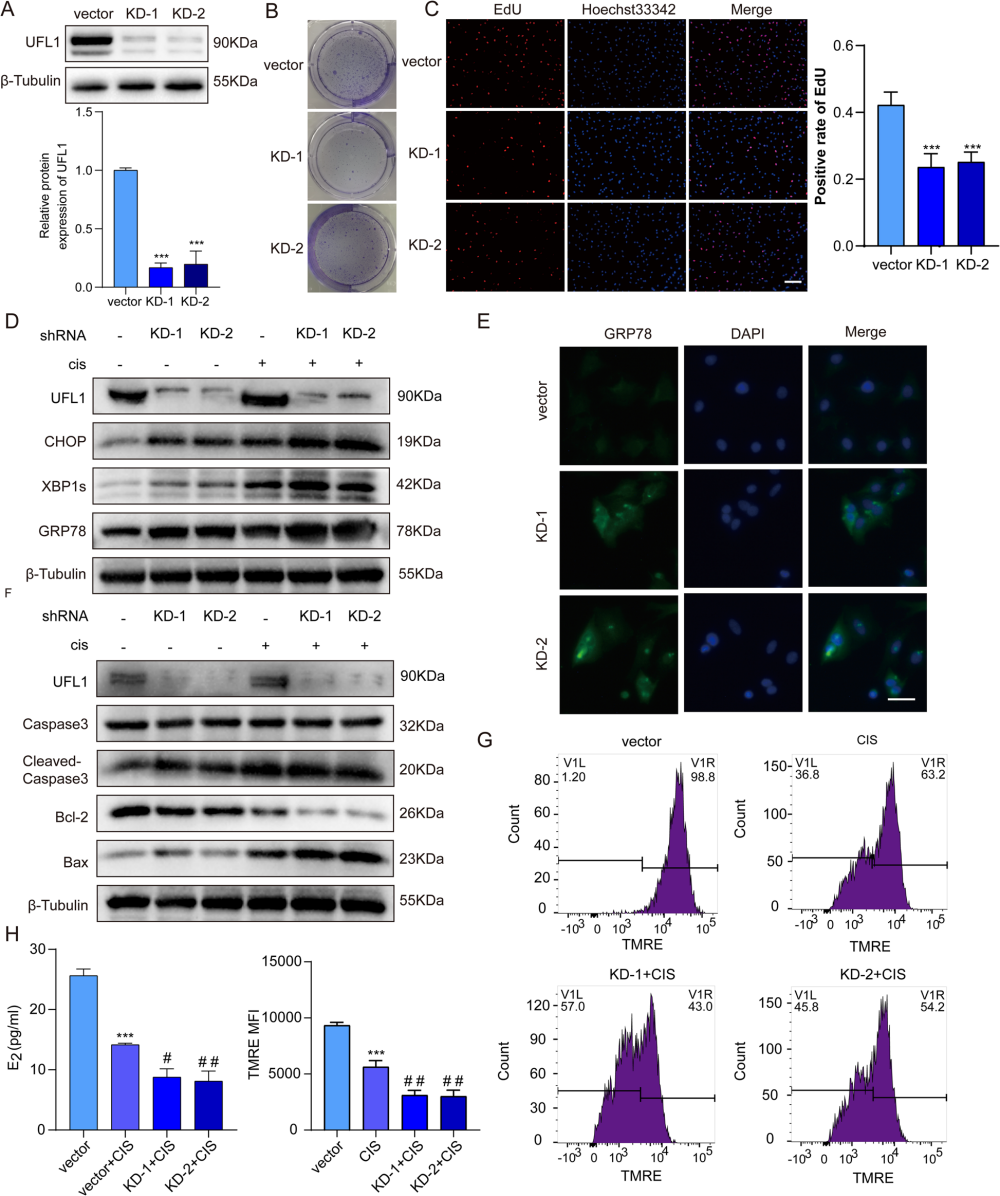
UFL1 depletion aggravates cisplatin-induced ER stress and apoptosis in GCs, with lower MMP and increased mitochondrial dysfunction. **A** The knock down efficiency of UFL1 shRNA. **B** UFL1 knockdown attenuated GCs colony formation. (**C**) The proliferative activity of GCs was evaluated by the EdU staining positive cell rate. Bar, 100 μm. **D** Changes in the protein expression of UFL1, GRP78, XBP1s and CHOP. (**E**) IF was used to detect the expression of GRP78 between the control and UFL1 knockdown GCs. Bar, 50 μm. **F** Changes in UFL1, BAX, BCL-2, cleaved caspase-3 and caspase-3 protein levels. **G** The mitochondrial membrane potential was measured by TMRE staining and measured by flow cytometry. **H** E_2_ secretion from GCs was detected by ELISA. *n* ≥ 3 for each group. * *p* < 0.05; ** *p* < 0.01; *** p < 0.001 compared with the control group. # *p* < 0.05 ## *p* < 0.01; ### *p* < 0.001 compared with the cisplatin treatment group

To confirm whether UFL1 is involved in ER stress and apoptosis under cisplatin treatment, we knocked down the expression of UFL1 with shRNA in GCs (Fig. 3A). Compared with the control group, the UFL1 knockdown groups exhibited weaker GC proliferation (Fig. 3B, C) and simultaneously enhanced expression of the ER stress proteins GRP78, XBP1s and CHOP (Fig. 3D, E), indicating that UFL1-depleted cells were more vulnerable to ER stress and apoptosis. As shown in Fig. 3D and 3F, compared with those in the CIS group, the levels of GRP78, XBP1s and CHOP were significantly increased (Figure S1J) in the UFL1 knockdown + cisplatin group and a similar trend was observed in the ratios of BAX/BCL-2 and cleaved caspase 3/caspase 3 (Figure S1K). The decrease in mitochondrial membrane potential (MMP) is a landmark event in the early stage of apoptosis. The evaluation of MMP was performed with TMRE, where the high potential is indicated by strong fluorescent intensity. Flow cytometry analysis showed that UFL1 deficiency further weakened MMP after cisplatin treatment (Fig. 3G). The observed changes in E_2_ concentration also verified the above results (Fig. 3H). Together, our experiments proved that UFL1 expression certainly correlates with ER stress and apoptosis induced by cisplatin.

### Overexpression of UFL1 resists cisplatin-induced ER stress and apoptosis

**Fig. 4:**
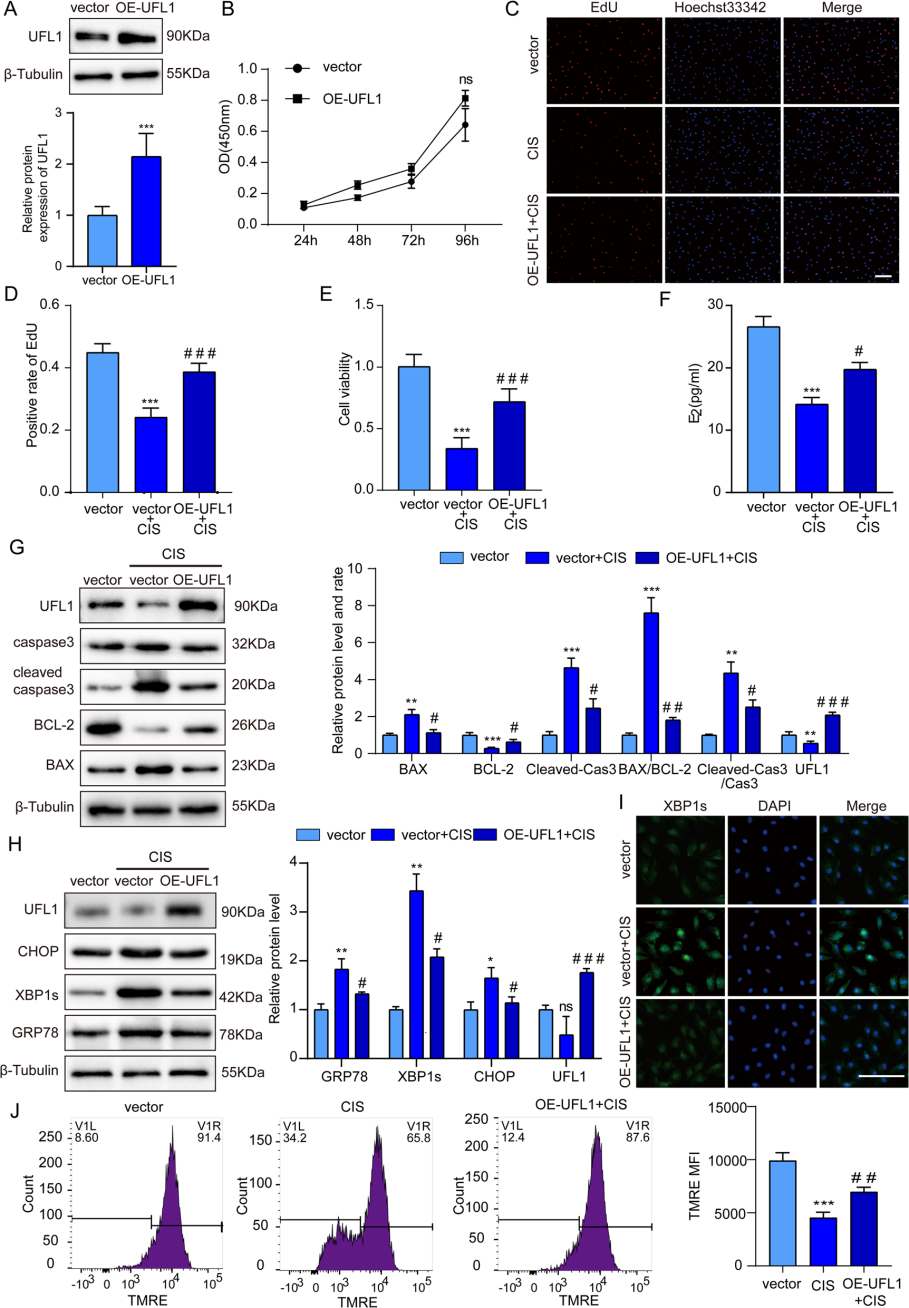
Overexpression of UFL1 in GCs alleviates cisplatin-induced apoptosis and ER stress. **A** Western blotting was used to detect UFL1 overexpression efficiency. **B** The cell growth curve of the UFL1 overexpression group. **C-D** The proliferative activity of GCs was evaluated by the EdU positive cell rate. Bar, 100 μm. **E** The cell viability of the UFL1 overexpression group was detected by CCK-8. **F** E_2_ levels in the supernatant of granulocyte cell culture. **G** Changes in UFL1, BAX, BCL-2, cleaved caspase-3 and caspase-3 protein levels with cisplatin treatment in UFL1 overexpressing GCs. **H** The protein levels of GRP78, XBP1s and CHOP with cisplatin treatment in UFL1 overexpressing GCs. **I** The expression of XBP1s in UFL1 overexpressing GCs treated with cisplatin was detected by immunofluorescence. Bar, 100 μm. **J **The mitochondrial membrane potential was measured by TMRE staining and measured by flow cytometry. *n* ≥ 3 for each group. * *p* < 0.05; ** *p* < 0.01; *** *p* < 0.001 compared with the control group. # *p* < 0.05; ## *p* < 0.01; ### *p* < 0.001 compared with the cisplatin treatment group

Furthermore, we attempted to investigate the protective effect of UFL1 against cisplatin treatment via overexpressing UFL1 (OE-UFL1) in GCs (Fig. 4A). Overexpression of UFL1 increased the growth rate of GC compared with the control group, but the P value was insignificant (Fig. 4B). The results of CCK-8 analysis and EdU staining showed that cellular viability and proliferation were increased after infection with UFL1 lentivirus particles (Fig. 4C-E), and E_2_ levels were increased simultaneously (Fig. 4F). Western blot results showed that overexpression of UFL1 decreased the ratios of BAX/BCL-2 and cleaved caspase 3/caspase 3, and downregulated the expression of Grp78, XBP1s and CHOP (Fig. 4H-I). In addition, TMRE staining showed that the level of MMP was higher in the OE-UFL1 group than the cisplatin treatment group (Fig. 4J). Overall, the overexpression of UFL1 can protect GCs from cisplatin-induced ER stress and apoptosis.

### The loss of UFL1 causes ovarian follicular atresia

**Fig. 5:**
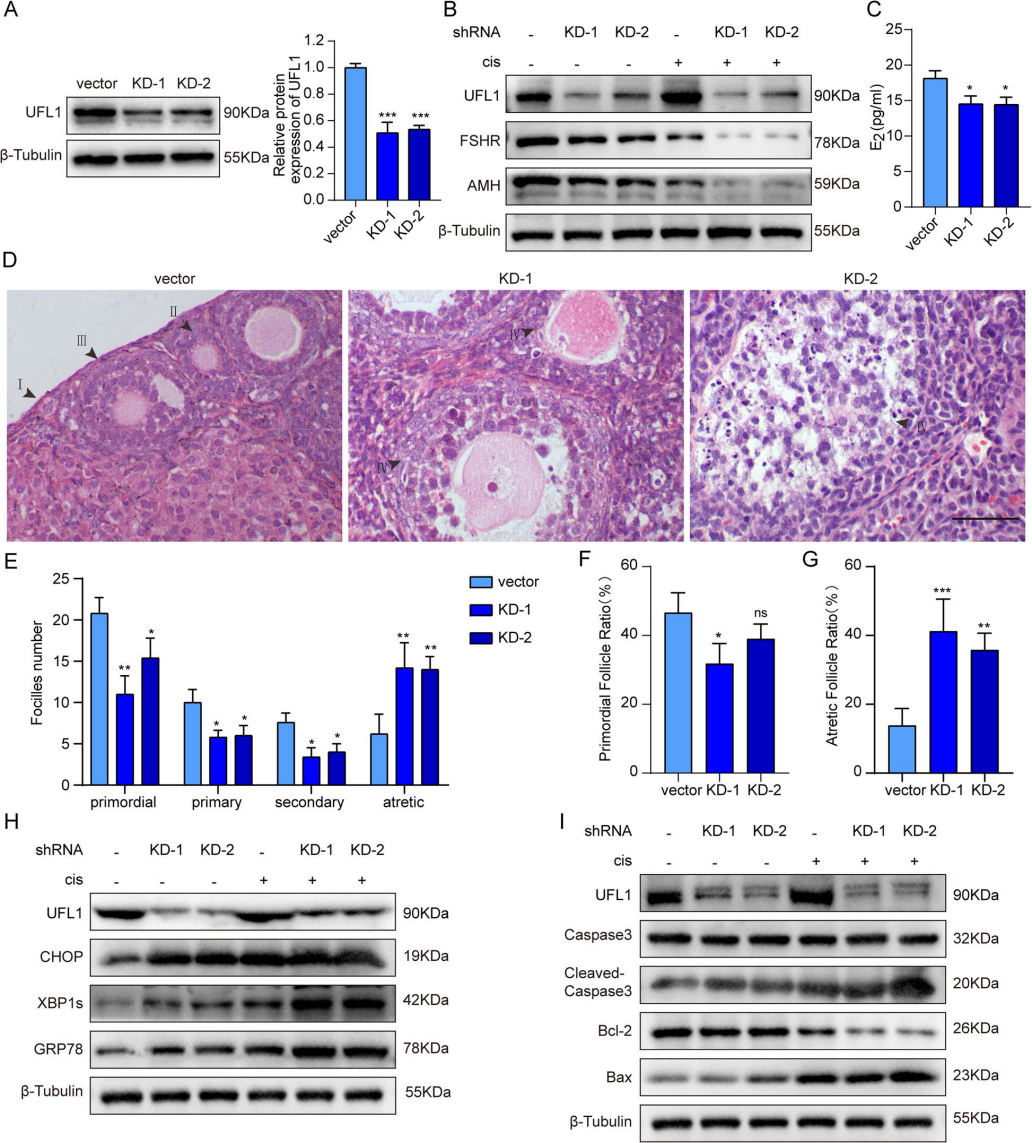
Loss of UFL1 led to activation of ovarian ER stress, increased atretic follicles, and decreased ovarian function. **A** Changes in the protein level of UFL1 in UFL1 knockdown ovaries. **B** Changes in the protein levels of UFL1, FSHR and AMH. **C** E_2_ levels in ovarian homogenate after UFL1 knockdown. **D-E** Follicles were observed after HE staining, and follicles of all stages were in tissue slices. Bar, 50 μm. Primordial follicle (I), primary follicle (II), secondary follicle (III), atretic follicle (IV). (**F, G**) The rate of follicle atresia and the primordial follicular rate vs. the total number of follicles in each group. **H** Changes in UFL1 protein and the ER stress markers GRP78, XBP1s and CHOP. **I** Changes in the proteins UFL1, BAX, BCL-2, cleaved caspase-3 and caspase-3. *n* ≥ 3 for each group. * *p* < 0.05; ** *p* < 0.01; *** *p* < 0.001 compared with the control group. # *p* < 0.05; ## *p* < 0.01; ### *p* < 0.001 compared with the cisplatin treatment group

To evaluate the role of UFL1 in maintaining ovarian function, we cultured ovaries with shRNA lentivirus particle suspension in vitro. As shown in Fig. 5A, we successfully knocked down the expression of UFL1 in the ovaries. The levels of FSHR and AMH indicate ovarian reserve function. Our results showed that their levels were reduced significantly after UFL1 knockdown (Fig. 5B, Figure S2A), and the concentration of E_2_ was also decreased to a certain extent (Fig. 5C). Compared with the control group, atretic follicles were newly observed, while primitive follicles were decreased, in the UFL1 knockdown group (Fig. 5D-G). Similar to the trend in UFL1 knockdown GCs, ER stress markers also showed an obvious increase in the ovarian UFL1 knockdown group (Fig. 5H). In addition, we examined UFL1 shRNA ovaries treated with cisplatin. Compared with the CIS group, UFL1 knockdown + CIS exhibited increased protein levels of GRP78, XBP1s and CHOP (Fig. 5H, Figure S2B). As a result, the expression ratios of the apoptotic proteins BAX/BCL-2 and cleaved caspase 3/caspase 3 were obviously increased in the combination group compared with the cisplatin-only group (Fig. 5I, Figure S2C), and FSHR and AMH showed a uniform trend (Fig. 5B, Figure S2A). Above all, these data indicate that the knockdown of UFL1 in ovaries induced follicular dysfunction, atresia and a decline in number, suggesting that UFL1 might play a crucial role in maintaining ovarian function.

### *UFL1 alleviates POF induced by Cisplatin *in Vitro

**Fig. 6:**
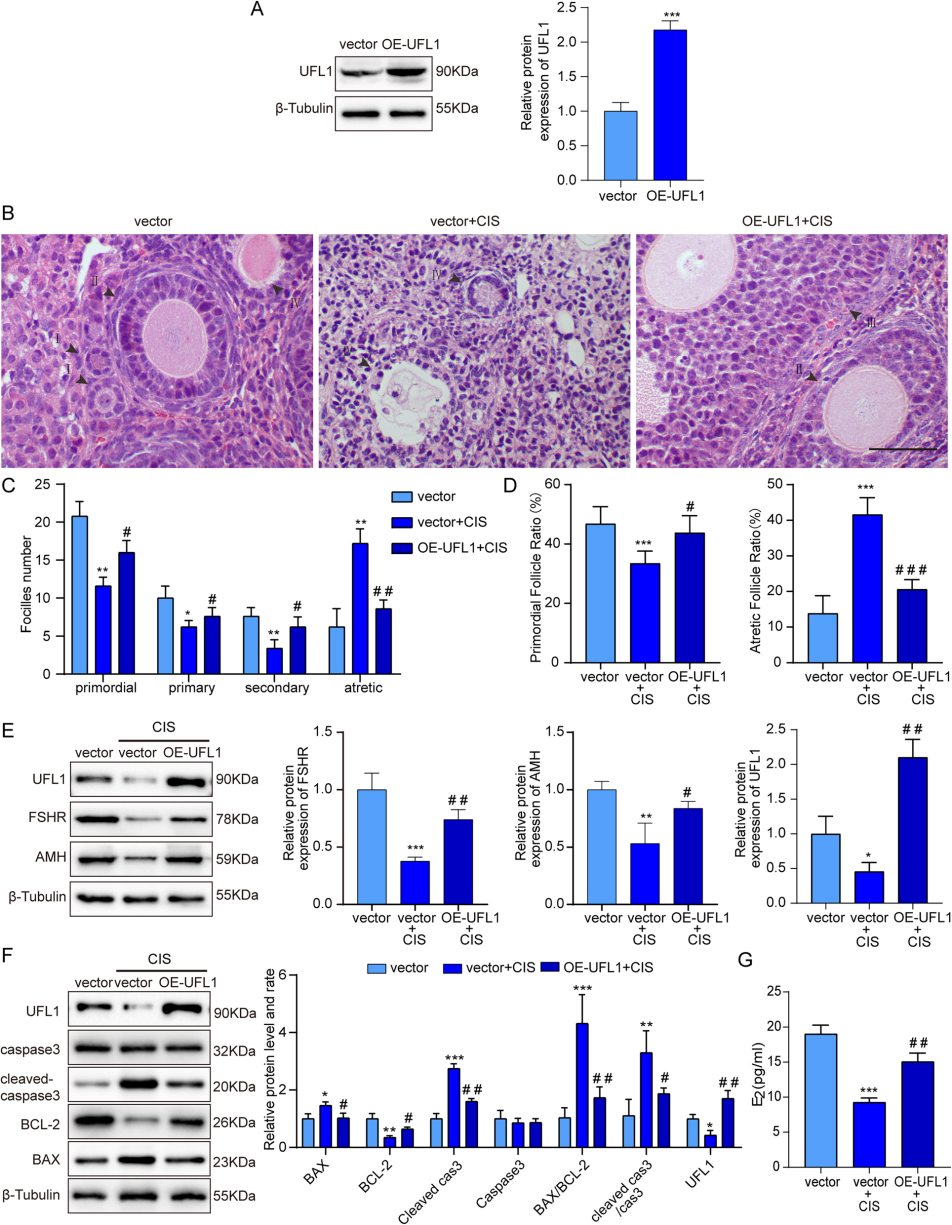
Overexpression of UFL1 in cultured ovaries alleviates apoptosis and dysfunction induced by cisplatin. **A** The efficiency of UFL1 overexpression was detected by western blot. **B** Follicles were observed after HE staining and follicles of all stages were counted in tissue slices. Bar, 20 μm. Primordial follicle (I), primary follicle (II), secondary follicle (III), atretic follicle (IV). **C, D** The rate of follicle atresia and the primordial follicular rate vs. the total number of follicles in each group. **E** Changes in UFL1, FSHR and AMH protein levels with cisplatin treatment in UFL1 overexpressing ovaries. **F** Changes in the BAX, BCL-2, cleaved caspase-3 and caspase-3 proteins in UFL1-overexpressing ovaries treated with cisplatin. *n* ≥ 3 for each group. * *p* < 0.05; ** *p* < 0.01; *** *p* < 0.001 compared with the control group. # *p* < 0.05; ## *p* < 0.01; ### *p* < 0.001 compared with the cisplatin treatment group

Next, we cultured ovaries in the cisplatin group with OE-UFL1 lentivirus particles. The efficiency of OE-UFL1 is shown in Fig. 6A. After 7 days of cultivation, the number of atretic follicles decreased and the number of primordial follicles increased compared with the numbers in the cisplatin-only group (Fig. 6B-D). Simultaneously, there was increased protein expression of AMH and FSHR (Fig. 6E). Furthermore, the protein levels of BAX and cleaved caspase-3 were decreased, and the level of BCL-2 was increased, in the OE-UFL1 group (Fig. 6F), and the ELISA results showed an increase in E_2_ concentration (Fig. 6G). Taken together, our data suggest that the overexpression of UFL1 can mitigate POF induced by cisplatin and augment follicle number to some extent.

## Discussion

Recent studies have confirmed that UFL1 plays a crucial role in biological processes, such as cell proliferation, differentiation and embryonic development [30–33], but the function of UFL1 in POF has not been explored. In this study, we revealed for the first time that UFL1 is expressed in GCs, oocytes and stromal cells in ovarian tissues (Fig. 1C-D). Comparing the expression of UFL1 in the ovaries at different developmental stages, it was found that the level of UFL1 increased significantly during follicular development, but decreased in aging ovaries (Fig. 1A). Follicular development is accompanied by the proliferation and differentiation of GCs, which implies plentiful protein synthesis and posttranslational modification (PTM) [34, 35]. Therefore, we speculated that the increased expression of UFL1 contributes to the maintenance of ER homeostasis in GCs, and provides a stable internal environment for follicular development. We detected the UFL1 protein level in a POF model, and the results showed that UFL1 was decreased in POF mice, which supports our speculation (Fig. 1B). Next, we tested ER stress specific molecules in the POF model and found that the levels of GRP78 and XBP1s were decreased (Fig. 2E, F). Interestingly, the expression of CHOP, which can activate the apoptotic pathway, was obviously increased (Fig. 2E, F). Previous studies have reported that ER stress can induce the expression of GRP78, XBP1s and other ER molecular chaperones to produce protective effects and trigger endogenous cell apoptosis, ultimately affecting outcomes such as adaptation, injury or apoptosis in stressed cells [36, 37]. Therefore, we hypothesized that the mechanism of POF caused by cisplatin occurs through severe ER stress, which eventually leads to the apoptosis of GCs, follicular atresia and ovarian dysfunction.

ER stress is a transient and dynamic process, and the molecular markers of ER stress are highly susceptible to other factors [38, 39]. To avoid stimulation of ER homeostasis during the primary isolation and culture of GCs, we treated normal GCs with cisplatin in vitro to replace primary POF GCs. The data showed that the level of UFL1 increased within 12 h in GCs treated with 20 μM cisplatin, and the changes in GRP78 and XBP1s showed the same trend as UFL1, while CHOP increased with prolonged treatment time (Fig. 2G, H). Interestingly, the expression of UFL1- and ER- specific markers in GCs decreased 24 h after high-concentration cisplatin (20 μM) treatment, which was the same pattern as was observed in POF ovaries (Fig. 2C-H). Cells accumulate a large number of misfolded proteins after cisplatin treatment, resulting in ER stress. Studies have proven that mild and transient ER stress can be alleviated by activating the UPR pathway while severe and continuous ER stress can induce the apoptotic pathway [40]. In this study, our data also confirm that cisplatin causes apoptosis in GCs by triggering severe ER stress. To verify whether UFL1 plays a protective role in ER stress, we knocked down and overexpressed UFL1 in GCs and ovaries respectively (Fig. 3A, Fig. 4A, Fig. 5A, Fig. 6A). The results showed that OE-UFL1 alleviated cisplatin-induced ER stress and apoptosis, reduced the number of atretic follicles and improved ovarian function (Fig. 4 and Fig. 6). Conversely, knockdown of UFL1 aggravated cisplatin damage (Fig. 3 and Fig. 5). In summary, our results indicate that UFL1 plays a protective effect, promoting GC survival and follicular number and protecting against follicle atresia, by alleviating ER stress and apoptosis.

In recent years, studies have shown that UFL1 can protect cells, such as bovine mammary epithelial cells, goat endometrial epithelial cells and human osteoarthritis chondrocytes, from LPS stimulation [7, 30, 41]. In this study, we demonstrated that UFL1 protects GCs from cisplatin damage by relieving ER stress. However, more research is needed to explore how UFL1 regulates ER stress. Walczak et al. discovered ribosomal RPL26 as a novel substrate of UFL1 conjugation, and the UFMylation of RPL26 is linked with ER homeostasis [42, 43]. Studies have shown that UFL1 first forms a receptor complex with C53 and DDRGK1, and then DDRGK1 recruits UFL1 to the ER surface for UFMylation-dependent ER autophagy [44]; their binding is necessary for the DDRGK1 UFMylation process [45, 46]. The C53 protein is an ER autophagy receptor, and UFL1 and DDRGK1 are codelivered to vacuoles with C53 and are essential for C53-mediated autophagy [46]. Therefore, the above studies suggest that UFL1 alters ER homeostasis through the ER autophagy pathway. In addition, UFL1 may induce ER stress by activating ferroptosis. As an important molecule in ferroptosis, P53 can be covalently modified by UFL1 and depletion of UFL1 can decrease P53 stability [47]. A related study has shown that the activation of ferroptosis induces an increase in ER stress [48]. Thus, UFL1 knockout may activate ferroptosis and induce ER stress by regulating P53 activity. In addition, the most recent research has reported that the protein stability of SLC7A11, which is a crucial ferroptosis regulator, can be reduced by inhibiting its UFMyltion [49]. Thus, we speculate that UFL1 deletion may reduce the UFMylation of SLC7A11 to activate ferroptosis-related ER stress.

In this study, we focused on whether UFL1 can relieve POF induced by cisplatin. The results indicated that UFL1 alleviates ovarian dysfunction and GC apoptosis by reducing ER stress in GCs, but more in vivo experiments are needed to evaluate the potential function of UFL1 as a target to alleviate ovarian aging and prevent POF caused by chemotherapy drugs. In conclusion, our study proved that UFL1 alleviated cisplatin-induced GC apoptosis and ER stress, providing a new strategy and perspective for preventing ovarian damage caused by chemotherapy drugs.

## Conclusions

In conclusion, our study proved that UFL1 alleviated cisplatin-induced GC apoptosis and ER stress, providing a new strategy and perspective for preventing ovarian damage caused by chemotherapy drugs.

## Supplementary Information

**Additional file 1.** 

**Additional file 2.** 

## Data Availability

All data generated through this study are included in this article.

## Abbreviations

UFL1: Ubiquitin-like modifier 1 ligating enzyme 1
ER stress: Endoplasmic Reticulum Stress
POF: Premature Ovarian Failure
GCs: Granulosa Cells
FSH: Follicle-Stimulating Hormone
E_2_: Estrogen
PERK: Protein kinase-like Endoplasmic Reticulum Kinase
UPR: The Unfolded Protein Response
P53: Transformation Related Protein 53
GRP78: Glucose Regulated Protein 78
XBP1s: The spliceosome of X-box Binding Protein 1
CHOP: C/EBP Homologous Transcription Factor
ATF4: Activating Transcription Factor 4
BCL-2: B-cell lymphoma-2
BAX: BCL2-Associated X
Cleaved Cas3: Cleaved Caspase-3
Cas3: Caspase3
AMH: Anti- Mullerian hormone
CIS: Cisplatin
FSHR: Follicle-Stimulating Hormone Receptor
DDRGK1: DDRGK domain containing 1
C53: CDK5 regulatory subunit associated protein 3
SLC7A11: Solute carrier family 7 (cationic amino acid transporter, y + system), member 11
